# Use of Artificial Neural Network in Determination of Shade, Light Curing Unit, and Composite Parameters' Effect on Bottom/Top Vickers Hardness Ratio of Composites

**DOI:** 10.1155/2018/4856707

**Published:** 2018-11-12

**Authors:** Hacer Deniz Arısu, Evrim Eligüzeloglu Dalkilic, Fehime Alkan, Sebnem Erol, Mine Betul Uctasli, Alican Cebi

**Affiliations:** ^1^Gazi University Faculty of Dentistry, Department of Restorative Dentistry, Ankara, Turkey; ^2^Bezmialem Vakıf University Faculty of Dentistry, Restorative Dentistry, Istanbul, Turkey; ^3^Yildiz Technical University, Faculty of Mechanical Engineering, Department of Mechanical Engineering, Heat and Thermodynamics Division, Istanbul, Turkey

## Abstract

**Objective:**

To assess the influence of light emitting diode (LED) and quartz tungsten halogen (QTH) light curing unit (LCU) on the bottom/top (B/T) Vickers Hardness Number (VHN) ratio of different composites with different shades and determination of the most significant effect on B/T VHN ratio of composites by shade, light curing unit, and composite parameters using artificial neural network.

**Method:**

Three composite resin materials [Clearfil Majesty Esthetic (CME), Tetric N Ceram (TNC), and Tetric Evo Ceram (TEC)] in different shades (HO, A2, B2, Bleach L, Bleach M) were used. The composites were polymerized with three different LED LCUs (Elipar S10, Bluephase 20i, Valo) and halogen LCU (Hilux). Vickers hardness measurements were made at a load of 100 g for 10 sec on the top and bottom surfaces and B/T VHN ratio calculated. The data were statistically analyzed with three-way ANOVA and Tukey test at a significance level of 0.05. The obtained measurements and data were then fed to a neural network to establish the correlation between the inputs and outputs.

**Results:**

There were no significant differences between the B/T VHN ratio of LCUs for the HO and B shades of CME (p>0.05), but there were significant differences between the B/T VHN ratio of LCUs for shade A2 (p<0.05). No significant difference was determined between the B/T VHN ratio of LCUs for all shades of TNC (p>0.05). For TEC, there was no significant difference between the B/T VHN ratio of halogen and LED LCUs (p>0.05), but a significant difference was determined among the LED LCUs (p<0.05). The artificial neural network results showed that a combination of the curing light and composite parameter had the most significant effect on the B/T VHN ratio of composites. Shade has the lowest effect on the B/T VHN ratio of composites.

**Conclusion:**

The B/T VHN ratio values of different resin-based composite materials may vary depending on the light curing device. In addition, the artificial neural network results showed that the LCU and composite parameter had the most significant effect on the B/T VHN ratio of the composites. Shade has the lowest effect on the B/T VHN ratio of composites.

## 1. Introduction

The polymerization of resin-based composites is still generally based on light activation of camphorquinone (CQ) [1]. CQ absorbs light and takes the molecules to excited states. From there, radicals or other initiating species start the conversion of the oligomer blend to a polymeric cross-linked network [2]. However, CQ has some disadvantages, such as poor polymerization efficacy, which affects the low mechanical feature of the resin [3]. Additionally, CQ is a dense yellow mixture, thus, having a large amount of CQ in the resin formulation leads to unwanted yellowing of the polymerized resin [4].

Lucirin TPO is now used in some composites because it is completely colorless after the light curing reaction, and its polymers are less yellow than others in which only camphorquinone is used as a photoinitiator. When a bleached tooth needs to be restored, the reduction of discoloration related to the photoinitiator is clinically significant in order to obtain and maintain color in aesthetic restorations [5]. Moreover, an excellent polymerization condition cannot be obtained when using the light emitting diode (LED) light curing unit (LCU) to photoactivate materials containing photoinitiators that absorb energy from another wavelength [6–8]. On the other hand, the broad spectrum of the quartz tungsten halogen (QTH) LCU, extending up to the ultraviolet region (UV-A), can be an advantage to excite coinitiators that absorb shorter wavelengths [9].

The degree of conversion (DC) of dental resin composites is crucial in determining the physical/mechanical performance of the material and its biocompatibility. Strength, modulus, hardness, and solubility are directly related to the DC [2]. The DC of resin composites is extensively assessed indirectly by surface hardness measurements, using either Vickers or Knoop indentors, which can give a good determination of DC [10, 11]. Substantial surface microhardness of the restoration is one of the main requirements for restorations especially in posterior stress-bearing areas [12, 13].

Artificial neural networks have numerous applications in scientific and social applications and their predictive abilities can cause many problems. They possess great opportunities for problems without clear mathematical linkages between the inputs and outputs. While neural networks can successfully predict many problems, additional caution must be taken to develop meaningful neural network structures, since by nature, neural networks do not recognize the physical meaning of inputs and outputs. Inadvertently designed neural network structures may not have the required generalization ability when showing applicable results for their original training data [14].

The aim of this study is to assess the influence of three different LED LCUs and a conventional QTH LCU on the bottom/top (B/T) Vickers hardness (VH) ratio of different composites with different shades. Another aim is to determine which parameters among shade, type of composite and type of light cure device has the strongest effect on the B/T VHN ratio of composites using an artificial neural network. The null hypothesis tested was that different LCUs did not affect the B/T VHN ratio of different composites with different shades.

## 2. Material and Methods

In the present study, three different composite resin materials [Clearfil Majesty Esthetic (CME) (Kuraray, Osaka, Japan), Tetric N Ceram (TNC) (Ivoclar Vivadent AG, Schaan, Liechtenstein), and Tetric Evo Ceram (TEC) (Ivoclar Vivadent AG, Schaan, Liechtenstein)] with different shades were used (Table 1). For each tested material, 20 cylindrical specimens (2-mm-depth and 5-mm-diameter) were prepared using metallic molds. In order to obtain a flat polymerized surface, the specimens were covered on both sides with a polyester matrix strip and a thin, rigid microscope slide and photopolymerized with a conventional QTH (900-1100mW/cm^2^, 380- 500 nm ) (Hilux, Benlioğlu Dental Inc., Ankara, Turkey**)** or Elipar S 10 (1200mW/cm^2^, 430–480 nm ) (3M ESPE, St Paul, MN,USA) Bluephase 20i (1200 mW/cm^2^, 385-515 nm ) (Ivoclar Vivadent,Liechtenstein), Valo (1000mW/cm^2^, 395-480 nm )(Ultradent Products Inc., South Jordan, UT)] curing units (n=5). Photoactivation was performed by positioning the light-guide tip to be in contact with the glass slide on the top surface of the specimen. Each specimen was irradiated according to the manufacturers' instructions for 40 s with QTH and 20 s with LED curing units. The specimens were removed from the mold and stored in 100% humidity, at 37°C for 24 hours. VHN measurements were made using a HMV Microhardness Tester (Shimadzu, Japan) at a load of 100 g for 10 s on the top and bottom surfaces. Three indentations were made on the top (upper) and bottom (lower) surfaces for each specimen, and the VHN of each surface was recorded as the average of these readings. The B/T VHN ratio of composites was calculated. Three-way ANOVA was performed to compare the dependent variable B/T VHN ratios for the fixed factors of three different composite resins, four different LCUs, and five different shades and their interactions at a significance level of 0.05. Multiple comparisons were made with Tukey's post hoc test.

### 2.1. Artificial Neural Network Analysis

In this study, different neural networks were employed using Matlab and their performances were evaluated to find the most successful network structure. Three different inputs and six outputs were used in this study, as shown in Table 2. Since the inputs of the study were nonnumeric, the enumeration technique was employed to use the inputs with the numeric output values by giving a unique number to every component used in the study. All of the inputs and outputs were normalized in the neural networks' operations. Different numbers of hidden layer neurons and different training functions, namely, Levenberg-Marquardt, Bayesian regulation, scaled conjugate gradient, and resilient backpropagation, were employed to find the network structure with the best predictive performance. Tangent sigmoid transfer functions were used for both layers. The Nguyen-Widrow initialization function was used for weights and biases to minimize the computation time. In order to prevent overfitting, appropriate convergence criteria were selected, and the validation process was monitored during the training phase.

The performance of the tested networks was determined by using mean square error (MSE) and correlation coefficient (R) values, which were defined as follows:

(1) MSE=1n∑i=1fi−yi2

(2) R=∑ifi−f−yi−y−∑ifi−f−2∑iyi−y−2

In the equations above, *f*_*i*_, *y*_*i*_, *n*, and $\overset{-}{y}$ were defined as the predicted value, experimental value, pattern number, and mean value of the experimental values, respectively

## 3. Results

Three-way ANOVA results showed that there were significances for composites (p=0.001), LCUs (p=0.001), and composite*∗*LCU interaction (p=0.001). No significance was observed for shade (p=0.328), composite*∗*shade interaction (p=0.807), shade*∗*LCU interaction (p=0.364), and composite*∗*shade*∗*LCU interaction (0.531). (Table 3)

Tukey post hoc test showed that there are significant differences between all composite materials (p<0.05). There was no significant difference between QTH LCU and Bluphase 20i (p=0.576) but there were significant differences between all other LCUs (p<0.05). When the shades were compared, there was no significant difference between B2 and HO, B2 and A2, A2 and HO, and M and L shades (p>0.05) but there were significant differences between all other shades (p<0.05). Multiple comparisons for composite, shade and LCUs are given in Tables 4–6, respectively.

When we split our data according to the B/T VHN ratios of the tested composite resin materials, the results are shown in Tables 7–9.  Table 7 shows B/T VHN ratios of Clearfil Majesty Esthetic (CME). There were no significant differences between the LCUs for the HO and B2 shades of CME (p>0.05), but there was a significant difference between the LCUs for shade A2 (p<0.05). Hilux gave the lowest B/T VHN ratio for A2, and Elipar showed the highest B/T VHN ratio. No significant difference was determined between shade HO, B, and A2.  Table 8 shows B/T VHN ratios for Tetric N Ceram Bleach (TNC). No significant difference was determined between the LCUs for all shades of TNC Bleach (p>0.05). No significant difference was determined between the Shade M and Shade L (p>0.05).  Table 9 shows the B/T VHN ratios for Tetric Evo Ceram (TEC). There were no significant differences between the halogen and LED LCUs for both shades of TEC (p>0.05). When the LED LCUs were compared, Bluephase 20i LCU showed significantly higher B/T VHN ratio values than Elipar did (p<0.05). There was no significant difference between the Bluephase 20i and Valo groups (p>0.05). When the shades were compared no significant difference was determined between Shade M and Shade L.

### 3.1. Artificial Neural Network Results

**Figure 1:**
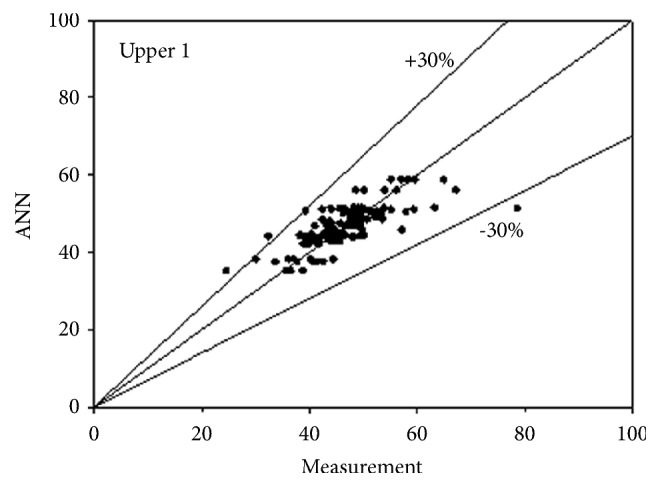
Predictability of ANN model for upper side according to measurement 1.

**Figure 2:**
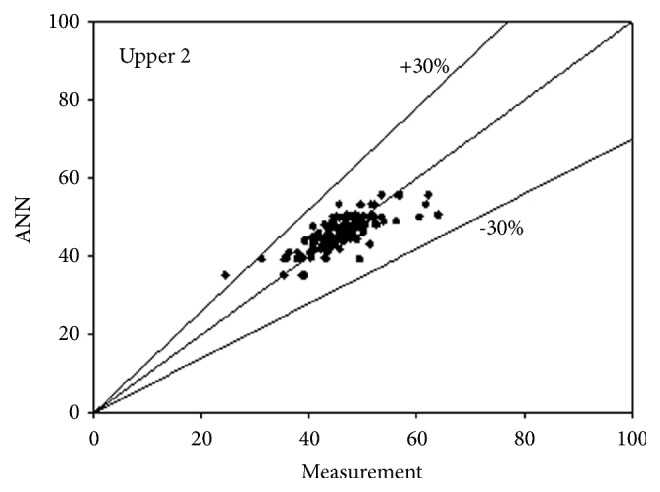
Predictability of ANN model for upper side according to measurement 2.

**Figure 3:**
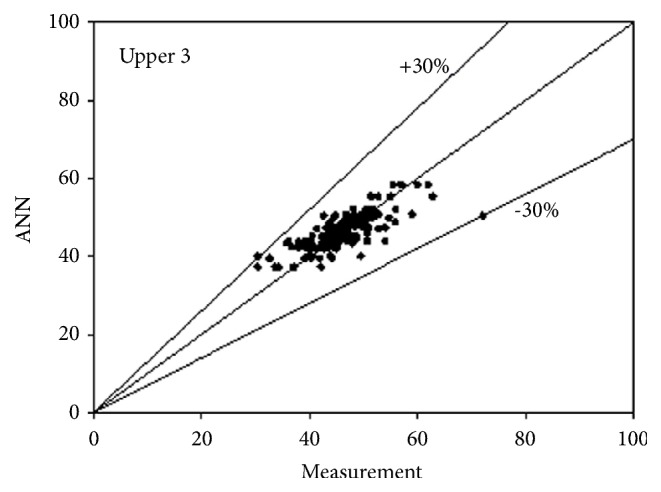
Predictability of ANN model for upper side according to measurement 3.

**Figure 4:**
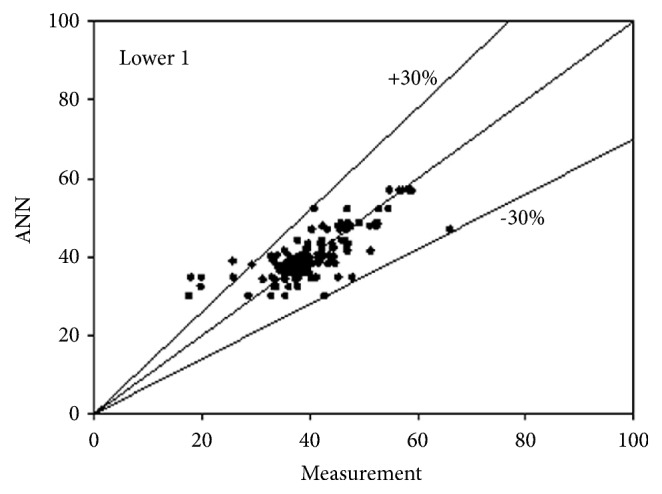
Predictability of ANN model for lower side according to measurement 1.

**Figure 5:**
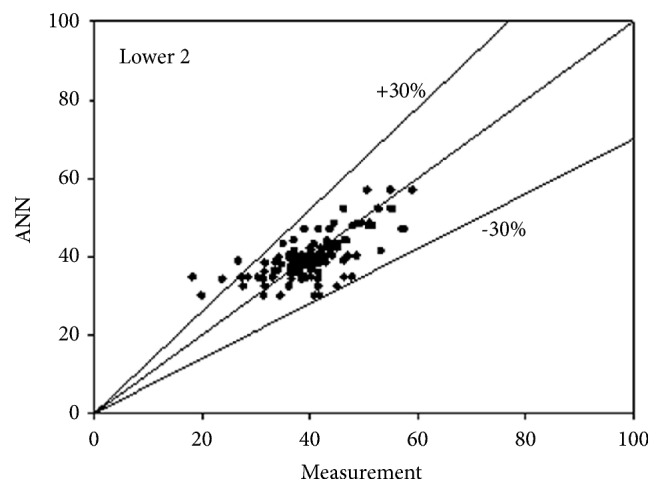
Predictability of ANN model for lower side according to measurement 2.

**Figure 6:**
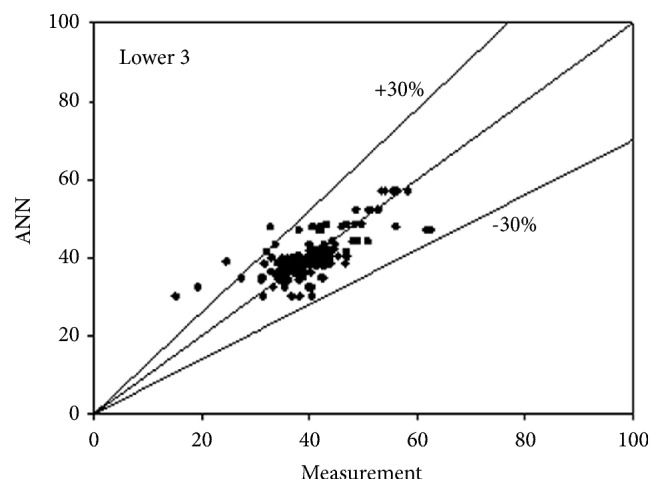
Predictability of ANN model for lower side according to measurement 3.

The best network structure to fit the data had 10 neurons in its hidden layer. Bayesian regulation was employed as the training function for the network. The MSE value of the network was 0.0373. The R values for the upper 3 and lower 3 outputs were 0.7487, 0.7890, 0.7721, 0.7674, 0.7674, and 0.7674, respectively. All figures, in subsequent chapters, as shown in Figures 1, 2, and 3 and Figures 4, 5, and 6, were drawn by using this neural network.

Dependency analysis was conducted to find the most influential parameter over the problem's outputs. The problems' inputs were fed to the neural network independently, and their performance was observed to reveal their impact on the outputs. The obtained results can be seen in Table 10. When single inputs were compared, curing light had the most significant effect, because it had the highest R values than the other two inputs. Shade has a lowest effect on the B/T VHN ratio of composites. A combination of composite and curing light inputs resulted in the highest R value among all of the combinations and had the most significant effect.

## 4. Discussion

In the present study, the B/T VHN ratio of different composites were determined when using different shades and when polymerized with different LCUs. The results showed that the B/T VHN ratios of the different composite materials with different shades varied depending on the light curing device used. Thus, the null hypothesis was rejected.

In the present study, LCUs with different light intensities and wavelengths were used to polymerize composites. The results show that the HO and B2 shades of the CME composite specimens showed similar B/T VHN ratios when polymerized with different LCUs. However, A2 shades of CME showed higher B/T VHN ratio when polymerized with Valo and Elipar than when polymerized with Hilux. This may be because of the different composition and photoinitiator ratio of CME in different shades. However, exact photoinitiator ratios and composition of these materials in different shades were not obtained from the manufacturer.

The lower transmittance of the light results in a low DC and consequently low microhardness, which is strongly influenced by the resin's opacity and its filler contents. However, different shades of all composites used in the present study showed similar B/T VHN ratios when polymerized with the same LCU.

Alternative photoinitiators like Lucirin TPO and Ivocerin have recently been added to composite resins [15, 16]. Both of the composite resins (TEC, TNC) used in the present study contain CQ in combination with TPO and Ivocerin. Different from CQ, the wavelengths of light absorbed by TPO are 350-425 nm, compared to 370-460 nm for Ivocerin [8]. TEC showed higher B/T VHN ratios when polymerized with Bluephase 20i with a wide-band spectrum of 385 to 515 nm than Elipar did with a narrow-band spectrum of 430 to 480 nm. The variation between the B/T VHN ratios with different LED LCUs may be because of the difference in wavelengths of the LCUs and the photoinitiator used in the composite resin. Because the LCUs achieved acceptable polymerization of a composite resin when the wavelengths of light absorbed by the photoinitiator fully or partially overlap the radiation spectrum of the LCU when used for polymerization. On the other hand, TNC showed similar B/T VHN ratio when polymerized with different LCUs. Although TNC and TEC have the same photoinitiator type and similar volume filler loading, different B/T VHN ratios obtained from different LCUs may be because of the different photoinitiator ratios of these materials. TEC has 48.5 wt % inorganic filler like glass, ytterbium trifluoride, and mixed oxide and 34.0 wt% prepolymer. Although TNC has similar filler loading with TEC, it has 63.5 wt% inorganic filler and only 17.0 wt % prepolymer. While the filler ratio of CME is higher than that of TNC and TEC, this material does not contain any prepolymer in its filler loading. When composite resin materials compared there is a significant difference between B/T VHN ratios of these three materials. TEC has the highest B/T VHN ratio followed by TNC and CME, however the filler loading (wt%) of these three materials are vice versa. CME has the highest filler loading followed by TNC and TEC. The difference between B/T VHN ratios may be attributed to the difference in the distribution of prepolymer filler loading of these three materials.

It is well proven that neural networks are suitable for complex problems that require extensive mathematical modelling. They can also be used on nonnumerical data which allows vast application areas such as advertisement, medicine, and sociology to use the extensive computation capability for generalization and prediction purposes. However special care must be taken when analyzing neural networks since network results would be accurate only as the size of the training data set. Therefore, data set must be selected such that the entire variable range is well presented, and necessary amount of data is present. As a result, this necessity requires extensive amount of data or experiments for a proper neural network training. Although satisfactory results and good generalization capability can be obtained, neural network should not be considered as a mathematical model by any means. This limits the use of neural network since the nature of the problem can not be solely determined from the network. It is also well known that the neural networks tend to give unsatisfactory results on data outside of the training data set or other data showing extreme characteristics. Acceptable results can be acquired for most of the complicated problems with carefully designed and trained networks however [17, 18].

For sufficient polymerization, three vital characteristics are essential for a LCU: adequate light output, adequate wavelength range of light, and efficient exposure time [19]. In this study, the artificial neural network results showed that the combination of composite and LCU parameter had the most significant effect on the B/T VHN ratio of the composites but shade has the lowest effect on the B/T VHN ratio. However, in the literature it is reported that shade plays an important role for light transmission through composites [20]. In a previous study, it was stated that resin composites of darker shades needed more energy for appropriate curing than those of lighter shades [21]. Another study reported that dark shades reduced light penetration to the bottom surfaces of composites, resulting in a reduction of depth of cure, bottom surface microhardness [22], so consequently this leads to a lover B/T VHN ratio. Similarly, Rodriques et al. [23] determined that shade had a greater influence on depth of cure of composites. They concluded that depth of cure of darker shades was lower than that of lighter shades. This study evaluated only the lighter shades of three different nanohybrid composites with similar inorganic filler loading, which is a limitation with respect to generalizing the conclusions. Studies should be done to evaluate the behavior of different shades and opacities of resin composites with different photoinitiators.

In a microhardness study, Sabatini [24] also reported that the surface hardness of the composites was affected by the type of LCU used for polymerization, although previous studies have demonstrated that the composite selection affects the performance of LCUs [15, 16, 19, 25]. Faria-E-Silva et al. [26] and Kramer et al. [27] decided that a number of factors can limit the depth of curing, including the type of resin composite, shade and translucency, increment thickness, distance from the tip of the LCU, postirradiation period, and size and distribution of filler particles. In the present study, although we used nanohybrid composites with similar inorganic filler loading, similar lighter shades with similar curing protocol the difference between B/T VHN ratios between composites and between LCUs can be attributed to different photoinitiator types and photoinitiator ratios of the composite resins and wide or narrow spectrum of the LCUs.

The efficiency of light curing techniques has often been assessed by depending on hardness measurements on the top and bottom surfaces of light-cured resin composite samples, and a bottom-to-top hardness ratio of 0.8 has been generally used as a standard for sufficient degree of cure [28, 29]. Applying this standard on the current results would suggest that some of the composites used in this study showed lower B/T VHN ratios when polymerized with different LCUs and did not produced acceptable hardness values. In addition to that some of the composites can be polymerized with QTH halogen and LED LCUs successfully. As is known, even a well polymerized composite resin can release residual monomers [30], elution of monomers from poorly polymerized resin from the bottom of a restoration is possible. Even though the current results were achieve in laboratory under ultimate conditions with high performance LCUs and probably the minimum distance that is clinically feasible, some of the groups did not have an acceptable B/T VHN ratio. Clinically, it is rarely probable to achieve perfect working conditions, and inadequate polymerization is to be expected.

## 5. Conclusion

Based on the current findings and within the limitations of this* in vitro* study, the B/T VHN ratios of different resin-based composite materials may vary depending on the light curing device. In addition, the artificial neural network results showed that the LCU and composite parameter had the most significant effect on the B/T VHN ratio of the composites. Shade has the lowest effect on the B/T VHN ratio of composites.

## Figures and Tables

**Table 1:** Materials and their composition.

Material Type	Organic Matrix	Inorganic Matrix	Photoinitiator	Shades
Clearfil Majesty	Bis-GMA,	Barium glass, silica	Camphorquinone	HO
Esthetic	TEGDMA	(85.5 wt%)	(468 nm)	A2
				B2

Tetric N Ceram	Bis-GMA-	Barium glass,	Lucirin TPO3	Bleach L
(Ivoclar	UDMA (15%),	ytterbium trifluoride,	(350-425 nm)	Bleach M
Vivadent AG,	Bis-EMA (3.8%)	oxides, silicon	+	
Schaan,		dioxide (63.5%)	Ivocerin	
Liechtenstein)		prepolymers (17%)	(370-460 nm)	
			+	
		81wt %, 55-57 vol%	camphorquinone	
			(468 nm)	

Tetric Evo	Bis-GMA,	Barium glass,	Lucirin TPO3	Bleach L
Ceram (Ivoclar	UDMA,	ytterbium trifluoride,	(350-425 nm)	Bleach M
Vivadent AG,	Bis-EMA	mixed oxide (48.5%)	+	
Schaan,	(16.8%)	prepolymers (34%)	Ivocerin	
Liechtenstein)			(370-460 nm)	
		80 wt%, 61 vol%	+	
			camphorquinone	
			(468 nm)	

**Table 2:** Inputs and outputs of the network.

Input 1	Input 2	Input 3	Outputs
Composite	Shade	Curing Unit	- Upper 1

- Clearfil Majesty esthetic	- B2	Hilux	- Upper 2

- Tetric N ceram bleach	- HO	Bluephase 20i	- Upper 3

- Tetric Evo ceram bleach	- A2	Valo	- Lower 1

	- M	Elipar S10	- Lower 2

	- L		- Lower 3

**Table 3:** Three-way ANOVA results to compare the dependent variable B/T VHN ratios for the fixed factors of three different composite resins, four different LCUs and five different shades and their interactions at a significance level of 0.05.

Dependent Variable: Bottom/top ratio					
Source	Type III Sum of Squares	df	Mean Square	F	Sig.
Corrected Model	28014,147^a^	27	1037,561	7,354	,000

Intercept	1213334,731	1	1213334,731	8600,098	,000

Composite	3076,818	1	3076,818	21,808	,000

Shade	491,862	3	163,954	1,162	,328

LCU	9541,274	3	3180,425	22,543	,000

Composite *∗* Shade	8,443	1	8,443	,060	,807

Composite *∗* LCU	5660,444	3	1886,815	13,374	,000

Shade *∗* LCU	1404,585	9	156,065	1,106	,364

Composite *∗* Shade *∗* LCU	312,873	3	104,291	,739	,531

Error	15801,388	112	141,084		

Total	1257150,267	140			

Corrected Total	43815,536	139			

a: R Squared =  ,639 (Adjusted R Squared =  ,552).

**Table 4:** Multiple comparisons of composite resin materials.

**Multiple Comparisons**						
Dependent Variable: Bottom/top ratio						
Tukey HSD						

(I) Composite	(J) Composite	Mean Difference (I-J)	Std. Error	Sig.	95% Confidence Interval	
					Lower Bound	Upper Bound

Clearfil	Tetric N Ceram	-8,3398^*∗*^	2,42456	,002	-14,0988	-2,5809
	Tetric Evo Ceram	-20,7431^*∗*^	2,42456	,000	-26,5021	-14,9841

Tetric N Ceram	Clearfil	8,3398^*∗*^	2,42456	,002	2,5809	14,0988
	Tetric Evo Ceram	-12,4033^*∗*^	2,65597	,000	-18,7119	-6,0946

Tetric Evo Ceram	Clearfil	20,7431^*∗*^	2,42456	,000	14,9841	26,5021
	Tetric N Ceram	12,4033^*∗*^	2,65597	,000	6,0946	18,7119

*∗*: the mean difference is significant at the 0,05 level.

**Table 5:** Multiple comparisons of shades.

**Multiple Comparisons**						
Dependent Variable: Bottom/top ratio						
Tukey HSD						

(I) Shade	(J) Shade	Mean Difference (I-J)	Std. Error	Sig.	95% Confidence Interval	
					Lower Bound	Upper Bound

B2	HO	3,4773	3,75611	,886	-6,9373	13,8920
	A2	6,5826	3,75611	,407	-3,8321	16,9972
	M	-10,3360^*∗*^	3,25289	,016	-19,3553	-1,3166
	L	-12,0404^*∗*^	3,25289	,003	-21,0597	-3,0210

HO	B2	-3,4773	3,75611	,886	-13,8920	6,9373
	A2	3,1053	3,75611	,922	-7,3094	13,5199
	M	-13,8133^*∗*^	3,25289	,000	-22,8326	-4,7939
	L	-15,5177^*∗*^	3,25289	,000	-24,5370	-6,4983

A2	B2	-6,5826	3,75611	,407	-16,9972	3,8321
	HO	-3,1053	3,75611	,922	-13,5199	7,3094
	M	-16,9186^*∗*^	3,25289	,000	-25,9379	-7,8992
	L	-18,6230^*∗*^	3,25289	,000	-27,6423	-9,6036

M	B2	10,3360^*∗*^	3,25289	,016	1,3166	19,3553
	HO	13,8133^*∗*^	3,25289	,000	4,7939	22,8326
	A2	16,9186^*∗*^	3,25289	,000	7,8992	25,9379
	L	-1,7044	2,65597	,968	-9,0687	5,6599

L	B2	12,0404^*∗*^	3,25289	,003	3,0210	21,0597
	HO	15,5177^*∗*^	3,25289	,000	6,4983	24,5370
	A2	18,6230^*∗*^	3,25289	,000	9,6036	27,6423
	M	1,7044	2,65597	,968	-5,6599	9,0687

*∗*: the mean difference is significant at the  ,05 level.

**Table 6:** Multiple comparisons of LCUs.

**Multiple Comparisons**						
Dependent Variable: Bottom-top ratio						
Tukey HSD						

(I) LCU	(J) LCU	Mean Difference (I-J)	Std. Error	Sig.	95% Confidence Interval	
					Lower Bound	Upper Bound

Halogen	Bluephase	-3,6400	2,83935	,576	-11,0451	3,7651
	Valo	-11,6836^*∗*^	2,83935	,000	-19,0887	-4,2785
	Elipar	-21,4835^*∗*^	2,83935	,000	-28,8887	-14,0784

Bluephase	Halogen	3,6400	2,83935	,576	-3,7651	11,0451
	Valo	-8,0437^*∗*^	2,83935	,028	-15,4488	-,6386
	Elipar	-17,8436^*∗*^	2,83935	,000	-25,2487	-10,4385

Valo	Halogen	11,6836^*∗*^	2,83935	,000	4,2785	19,0887
	Bluephase	8,0437^*∗*^	2,83935	,028	,6386	15,4488
	Elipar	-9,7999^*∗*^	2,83935	,004	-17,2050	-2,3948

Elipar	Halogen	21,4835^*∗*^	2,83935	,000	14,0784	28,8887
	Bluephase	17,8436^*∗*^	2,83935	,000	10,4385	25,2487
	Valo	9,7999^*∗*^	2,83935	,004	2,3948	17,2050

*∗*: the mean difference is significant at the  ,05 level.

**Table 7:** B/T VHN ratio for Clearfil Majesty Esthetic.

	Hilux	Bluephase 20i	Valo	Elipar S 10
B2	86.7 Aa	82.7 Aa	85.1 Aa	97.9 Aa
A2	68.6 Aa	76.3 ABa	88.7 Ba	92.5 Ba
HO	77.2 Aa	76.7 Aa	90.7 Aa	93.9 Aa

Capital letters show the significant differences between the light curing units; lower case shows the significant difference between the shades (p<0.05).

**Table 8:** B/T VHN ratio for Tetric N Ceram Bleach.

	Hilux	Bluephase 20i	Valo	Elipar S 10
Shade M	90.3 Aa	95.8 Aa	91.6 Aa	90.1 Aa
Shade L	91.9 Aa	91.3 Aa	96.4 Aa	97.5 Aa

Capital letters show the significant differences between the light curing unit; lower case shows the significant difference between the shades (p<0.05).

**Table 9:** B/T VHN ratio for Tetric Evo Ceram Bleach.

	Hilux	Bluephase 20i	Valo	Elipar S10
Shade M	87.4 ABa	96.1 Aa	89.8 ABa	80.8 Ba
Shade L	84.7 AB a	93.8 Aa	96.7 Aa	73.4 Ba

Capital letters show the significant differences between the light curing unit; lower case shows the significant difference between the shades (p<0.05).

**Table 10:** Dependency analysis of inputs.

Neural network inputs			R					
Composite	Shade	Curing Unit	Upper 1	Upper 2	Upper 3	Lower 1	Lower 2	Lower 3
X	X	X	0.7487	0.7890	0.7721	0.7674	0.7674	0.7674
X			0.2207	0.2191	0.2497	0.2654	0.2655	0.2654
	X		0.1299	0.0680	0.1493	0.1795	0.1795	0.1795
		X	0.4459	0.4808	0.5126	0.3927	0.3927	0.3928
X	X		0.2080	0.1702	0.2501	0.2452	0.2452	0.2452
X		X	0.6222	0.6549	0.6642	0.6494	0.6494	0.6494
	X	X	0.1039	0.0875	0.1538	0.2444	0.2444	0.2443

## Data Availability

The data used to support the findings of this study are available from the corresponding author upon request.

## References

[1] Miletic V., Santini A. (2012). Optimizing the concentration of 2,4,6-trimethylbenzoyldiphenylphosphine oxide initiator in composite resins in relation to monomer conversion. *Dental Materials*.

[2] Stansbury J. W. (2000). Curing dental resins and composites by photopolymerization. *Journal of Esthetic Dentistry*.

[3] Schroeder W. F., Vallo C. I. (2007). Effect of different photoinitiator systems on conversion profiles of a model unfilled light-cured resin. *Dental Materials*.

[4] Arikawa H., Takahashi H., Kanie T., Ban S. (2009). Effect of various visible light photoinitiators on the polymerization and color of light-activated resins. *Dental Materials*.

[5] Miletic V., Pongprueksa P., De Munck J., Brooks N. R., Van Meerbeek B. (2013). Monomer-to-polymer conversion and micro-tensile bond strength to dentine of experimental and commercial adhesives containing diphenyl(2,4,6- trimethylbenzoyl)phosphine oxide or a camphorquinone/amine photo-initiator system. *Journal of Dentistry*.

[6] Emami N., Söderholm K.-J. M. (2005). Influence of light-curing procedures and photo-initiator/co-initiator composition on the degree of conversion of light-curing resins. *Journal of Materials Science: Materials in Medicine*.

[7] Rahiotis C., Kakaboura A., Loukidis M., Vougiouklakis G. (2004). Curing efficiency of various types of light-curing units. *European Journal of Oral Sciences*.

[8] Price R. B. T., Felix C. A., Andreou P. (2005). Knoop hardness of ten resin composites irradiated with high-power LED and quartz-tungsten-halogen lights. *Biomaterials*.

[9] Dunn W. J., Bush A. C. (2002). A comparison of polymerization by light-emitting diode and halogen-based light-curing units. *The Journal of the American Dental Association*.

[10] Price R. B. T., Ehrnford L., Andreou P., Felix C. A. (2003). Comparison of quartz-tungsten-halogen, light-emitting diode, and plasma arc curing lights. *The Journal of Adhesive Dentistry*.

[11] Park S. H., Krejci I., Lutz F. (2002). Microhardness of resin composites polymerized by plasma arc or conventional visible light curing. *Operative Dentistry*.

[12] Mousavinasab S. M., Meyers I. (2011). Comparison of depth of cure, hardness and heat generation of LED and high intensity QTH light sources. *European Journal of Dentistry*.

[13] Moraes L. G. P., Rocha R. S. F., Menegazzo L. M., De Araújo E. B., Yukimitu K., Moraes J. C. S. (2008). Infrared spectroscopy: A tool for determination of the degree of conversion in dental composites. *Journal of Applied Oral Science*.

[14] Fausett L. (1994). *Fundamentals of neural networks, Architectures, Algorithms, and Applications*.

[15] Leonard D. L., Charlton D. G., Roberts H. W., Cohen M. E. (2002). Polymerization efficiency of LED curing lights. *Journal of Esthetic and Restorative Dentistry*.

[16] Uhl A., Sigusch B. W., Jandt K. D. (2004). Second generation LEDs for the polymerization of oral biomaterials. *Dental Materials*.

[17] Curry B., Moutinho L. (1993). Neural networks in marketing: modelling consumer responses to advertising stimuli. *European Journal of Marketing*.

[18] Bainbridge W. S. (1995). Neural network models of religious belief. *Sociological Perspectives*.

[19] Knežević A., Tarle Z., Meniga A., Šutalo J., Pichler G., Ristić M. (2001). Degree of Conversion and temperature rise during polymerization of composite resin samples with blue diodes. *Journal of Oral Rehabilitation*.

[20] Yu B., Lee Y.-K. (2008). Influence of color parameters of resin composites on their translucency. *Dental Materials*.

[21] Passos S. P., Kimpara E. T., Bottino M. A., Santos G. C., Rizkalla A. S. (2013). Effect of ceramic shade on the degree of conversion of a dual-cure resin cement analyzed by FTIR. *Dental Materials*.

[22] Aguiar F. H. B., Lazzari C. R., Lima D. A. N. L., Ambrosano G. M. B., Lovadino J. R. (2005). Effect of light curing tip distance and resin shade on microhardness of a hybrid resin composite.. *Brazilian Oral Research*.

[23] Rodriguez A., Yaman P., Dennison J., Garcia D. (2017). Effect of light-curing exposure time, shade, and thickness on the depth of cure of bulk fill composites. *Operative Dentistry*.

[24] Sabatini C. (2013). Comparative study of surface microhardness of methacrylate-based composite resins polymerized with light-emitting diodes and halogen. *European Journal of Dentistry*.

[25] Uhl A., Mills R. W., Vowles R. W., Jandt K. D. (2002). Knoop hardness depth profiles and compressive strength of selected dental composites polymerized with halogen and LED light curing technologies. *Journal of Biomedical Materials Research Part B: Applied Biomaterials*.

[26] Faria-e-Silva A. L., Fanger C., Nguyen L., Howerton D., Pfeifer C. S. (2017). Impact of material shade and distance from light curing unit tip on the depth of polymerization of composites. *Brazilian Dental Journal*.

[27] Krämer N., Lohbauer U., García-Godoy F., Frankenberger R. (2008). Light curing of resin-based composites in the LED era. *American Journal of Dentistry*.

[28] Flury S., Hayoz S., Peutzfeldt A., Hüsler J., Lussi A. (2012). Depth of cure of resin composites: is the ISO 4049 method suitable for bulk fill materials?. *Dental Materials*.

[29] Moore B. K., Platt J. A., Borges G., Chu T.-M. G., Katsilieri I. (2008). Depth of cure of dental resin composites: ISO 4049 depth and microhardness of types of materials and shades. *Operative Dentistry*.

[30] Tanaka K., Taira M., Shintani H., Wakasa K., Yamaki M. (1991). Residual monomers (TEGDMA and Bis-GMA) of a set visible-light-cured dental composite resin when immersed in water. *Journal of Oral Rehabilitation*.

